# Melatonin as a Harmonizing Factor of Circadian Rhythms, Neuronal Cell Cycle and Neurogenesis: Additional Arguments for Its Therapeutic Use in Alzheimer’s Disease

**DOI:** 10.2174/1570159X21666230314142505

**Published:** 2023-04-12

**Authors:** Mayuri Shukla, Bruno Vincent

**Affiliations:** 1 Institute of Molecular Biosciences, Mahidol University, Nakhon Pathom 73170, Thailand;; 2 Present Address: Chulabhorn Graduate Institute, Chulabhorn Royal Academy, 10210, Bangkok, Thailand;; 3 Institute of Molecular and Cellular Pharmacology, Laboratory of Excellence DistALZ, Université Côte d'Azur, INSERM, CNRS, Sophia-Antipolis, 06560, Valbonne, France

**Keywords:** Melatonin, circadian rhythm, cell cycle, Alzheimer’s disease, neurogenesis, therapeutic

## Abstract

The synthesis and release of melatonin in the brain harmonize various physiological functions. The apparent decline in melatonin levels with advanced aging is an aperture to the neurodegenerative processes. It has been indicated that down regulation of melatonin leads to alterations of circadian rhythm components, which further causes a desynchronization of several genes and results in an increased susceptibility to develop neurodegenerative diseases. Additionally, as circadian rhythms and memory are intertwined, such rhythmic disturbances influence memory formation and recall. Besides, cell cycle events exhibit a remarkable oscillatory system, which is downstream of the circadian phenomena. The linkage between the molecular machinery of the cell cycle and complex fundamental regulatory proteins emphasizes the conjectural regulatory role of cell cycle components in neurodegenerative disorders such as Alzheimer’s disease. Among the mechanisms intervening long before the signs of the disease appear, the disturbances of the circadian cycle, as well as the alteration of the machinery of the cell cycle and impaired neurogenesis, must hold our interest. Therefore, in the present review, we propose to discuss the underlying mechanisms of action of melatonin in regulating the circadian rhythm, cell cycle components and adult neurogenesis in the context of AD pathogenesis with the view that it might further assist to identify new therapeutic targets.

## 1 INTRODUCTION

Several theories have been postulated with respect to the pathogenesis of Alzheimer’s disease (AD), a neurodegenerative disorder presenting a very peculiar conformity in terms of initiation of pathology and symptomology. The neurodegenerative countenances befall years before the symptoms show up, with the formation of senile plaques and neurofibrillary tangles being contemplated as its characteristic pathological hallmarks. Given the prevalence of rhythmic anomalies during aging [1] and in neurodegenerative pathologies [2], the molecular circadian network directly influences the course of such states, thereby supporting the hypothesis that circadian disruption is one major symptom in neurodegenerative diseases [3]. Indeed, circadian rhythm upheaval is precisely involved in the etiopathogenesis and progression of AD, and alterations in this rhythm could conceivably presage the onset of dementia [4].

The zeitgebers-like light synchronizes the daily rhythms [5], which are generated in the suprachiasmatic nucleus (SCN) located in the ventral hypothalamus. Circadian rhythms are commanded by this principal pacemaker that controls the coordinated peripheral oscillators and regulates the physiological, biochemical, and behavioural activities. Studies have shown that apart from the hypothalamus, other regions in the brain, such as the hippocampus and the cortex, possess oscillators capable of generating circadian rhythms [6, 7]. The crosstalk between the circadian clock and cellular signalling thereby contributes to the synchronization of circadian oscillations and physiological homeostasis [8, 9].

It is interesting to point out that abnormal circadian rhythms are one of the characteristic features of not only AD but also of several other neurodegenerative diseases, including Parkinson’s disease (PD) and Huntington's disease (HD) [10, 11], one extremely important aspect of the fundamental function of circadian clocks being the regulation of proteostasis, where altered daily rhythms could significantly influence and enhance pro-neurodegenerative molecular aggregations [12].

Moreover, the occurrence of circadian rhythm disruptions before the onset of typical clinical symptoms of neurodegeneration suggests that such rhythmic alterations are early events that might also be a potential risk factor for developing AD and PD [13, 14]. The circadian system, therefore, could be a new diagnostic and therapeutic target in neurodegenerative disorders. As such, clinical trials become essential to determine whether circadian interventions could prevent or delay the onset of AD and related dementias [15] and if modulating the circadian state of brain cells susceptible to neurodegeneration could represent a possible anti-AD therapeutic track [16].

One additional important point is that adult neurogenesis, which is altered in AD [17], is under the control of the circadian system [18], thereby suggesting that restored neurogenesis could assist future innovative circadian rhythms-synchronizing treatments in alleviating AD-related cognitive symptoms.

The cell cycle is a highly regulated process that maintains a homeostatic balance between cell proliferation and cell death and that consists of four phases G1 (cell growth), S (DNA replication), G2 (cell growth) and M (mitosis), the progression of which is under the control of cell cycle regulators that coordinate the transition from one phase to another [19]. Cell cycle oddity is an early feature of AD that has been extensively evidenced in the aetiology of the disease as manifesting abnormal cell cycle re-entry that precedes the pathological hallmarks of AD [20-22]. Thus, the neuronal cell cycle re-entry in humanized Aβ plaque-producing AppNLF knock-in mice results in the development of subsequent AD-related pathological events, including tauopathy, neuroinflammation, brain leucocytes infiltration, DNA damage response and neurodegeneration [23].

Neurons generally remain in the G0 phase (quiescent state), but toxic insults and different kinds of stressors force neurons to exit G0 and induce cell cycle re-entry leading to cell death *via* apoptosis. Cell cycle reactivation in adult neurons is a sign of neurodegeneration [24] and central nervous system (CNS) lesions [25, 26], and the cell-cycle hypothesis of AD considers this disease a consequence of deregulation of the cell cycle in neurons. Although studies in rodents and primates have indicated that regulation of the cell cycle plays a crucial role in controlling area-specific neuron production [27], an aberrant reactivation of the cell cycle in these neurons initiates the complex process of apoptosis [28], which likely contributes to AD development [29, 30] and is associated with the cardinal hallmarks of AD including tau hyper phosphorylation and amyloid beta (Aβ) accumulation [31] along with consequent degeneration of neurons [32]. The mechanistic features of the involved cell cycle machinery would thus aid in understanding its physiological and pathogenic implications in the disease state [33].

Melatonin is a key output of the circadian clock, a circadian regulator and plays a major role in peripheral clock synchronization due to its strong circadian expression pattern. Under direct control from the SCN, the pineal gland synthesizes and releases melatonin into circulation in response to darkness, where its secretion is known to be suppressed by light [34]. Also, animal studies showed that retinal melatonin plays an important role in the regulation of retinal daily and circadian rhythms [35]. The most direct route by which melatonin can reach the SCN is considered to be *via* the cerebrospinal fluid of the third ventricle. Melatonin can also reach the pars tuberalis (PT) of the pituitary, another melatonin-sensitive tissue, *via* this route. Melatonin, in turn, acts on the SCN by directly influencing the circadian clock mechanisms and by controlling circadian rhythms in association with molecular timing genes [36]. The hypothalamic high-affinity G protein-coupled melatonin receptors MT_1_ and MT_2_ are involved in the regulation of circadian rhythms, suggesting clinical applications of melatonin receptor-targeting drugs [37]. Moreover, findings from preclinical and clinical studies revealed some roles of melatonin in the control of the circadian clock-associated genes, thus suggesting that the hormone could have beneficial effects in AD patients *via* an improvement of their circadian rhythms given that AD pathogenesis involves atrophic alterations in the brain resulting in circadian disruptions [38]. Indeed, melatonin treatment has beneficial effects in mild cognitive impairment (MCI) and AD patients with sleep disorders in improving sleep quality and in regulating the sleep/wake rhythm [39]. Melatonin has been recognized to alleviate pathogenic mechanisms in AD [40] by regulating several signalling pathways [41]. Recently, it has also been demonstrated that melatonin, by modulating specific pathways, might also play a role in cell cycle functions related to AD [42]. Moreover, a complete understanding of the interrelationship between AD and circadian rhythm disruption might concede for earlier identification of AD diagnostic [43]. Therefore, the present review emphasizes these two particular mechanisms, which can probably portend the onset of neurodegenerative diseases like AD and determine further clinical interventions.

## 2 MELATONIN, AGING AND AD

### 2.1 Melatonin and Normal Aging

It is well established that melatonin secretion is maximum during brain development (from childhood to adolescence) and declines gradually over the lifespan with very low production at an advanced age [44]. This drop in melatonin levels occurs due to dysfunction of the suprachiasmatic nucleus or of the neuronal pathways of transmission to the pineal gland. As a consequence, aging is tightly associated with a weakening of the circadian system. In addition, because melatonin is a highly pleiotropic regulatory molecule that not only acts through its antioxidant properties but also plays important physiological and pharmacological roles in the control of neuronal plasticity and neuroprotection, its drop in level is expected to reduce adult hippocampal neurogenesis [45]. Moreover, the loss of amplitude of melatonin rhythm in advanced age is both an indicator as well as a cause of age-related disturbances in the circadian pacemaker leading to chronobiological disorders [46]. The effect of melatonin on aging-associated pathologies, with an emphasis on data from aged organisms and senescence-accelerated animals, has been extensively and critically reviewed [47].

In this context, the chronobiotic efficacy of exogenous melatonin has been demonstrated in both experimental models and clinical studies with validated recommendations for treatment purposes [48] and slowing the process of aging [49] and neurodegenerative diseases [50]. Importantly, age-specific preventive clinical and therapeutic applications of melatonin in newborns, children and adults based on its physiological regulatory effects have been substantiated to better understand the short- and long-term effects of melatonin following its immediate or prolonged release [51]. Moreover, the influence of endogenous and exogenous melatonin on the adolescent brain, with specific reference to the evolution of brain structure and functions, sleep regulation, and modulation of behaviors in health or disease, has been well described [52] and a review on the ongoing clinical trials on the effects of melatonin on circadian rhythms in young adults with at-risk symptoms has been provided [53]. However, the dosage paradigm and duration of exposure is the key to a better prognosis [54]. Recently, the benefits and adverse events of melatonin use in the elderly have been extensively discussed [55].

### 2.2 Melatonin in AD and its Therapeutic Efficacy

Disruptions in melatonin levels in the CSF, blood, saliva and urine, which occur with age and are more pronounced in AD, have been recently overviewed and summarized extensively [56]. Moreover, studies assessing the effect of melatonin on transgenic models of AD and clinical evaluations of melatonin treatment of MCI and AD patients have been well documented [57]. Furthermore, clinical analysis of melatonin therapy in AD patients [58] and meta-analysis of randomized, double-blind, placebo-controlled trials of melatonin in AD pathology [59] gave insight into the therapeutic potential of melatonin and how the dosage regimen should be applied. Additional clinical investigations provide evidence to support the beneficial effects of melatonin on sleep disorders and cognitive deficits in AD patients [60].

On this point, it has been recently evidenced that sleep deprivation can be fatal due to an accumulation of reactive oxygen species (ROS) in the gut in drosophila and mice [61]. Given the important roles played by melatonin in sleep regulation as well as its antioxidant power, these findings certainly reinforce the validity of the use of melatonin for the reduction of sleep disturbances and oxidative stress observed in AD.

Besides, melatonin *via* its receptor activation can modulate the survival of newborn neurons in the adult hippocampus, making it the first known exogenously applicable substance with such specificity [62]. The beneficial therapeutic effects of melatonin for neurogenesis impairment and the associated underlying molecular mechanisms in aging and neurological disorders, including AD, have been comprehensively reviewed [40, 63]. Moreover, because melatonin stimulates early and late stages of neurodevelopment in the adult brain and significantly enhances memory and cognitive functions, its beneficial effects in positively regulating neurogenesis in AD are more than likely [42].

The clinical efficacy of melatonin intervention for cognitive function in AD and its clinical effectiveness for cognitive function in healthy subjects has been systematically reviewed based on a meta-analysis of randomized controlled trials with specific emphasis on the duration of melatonin intervention [64]. Recently, the developmental trends of melatonin therapies from preclinical studies for AD, the beneficial effects of melatonin on behavior in animal models of AD, and the clinical effects of melatonin on sleep, cognition, behavior, psychiatric symptoms, electroencephalography findings, and molecular biomarkers in patients with mild cognitive impairment and AD along with limitations of current melatonin therapies for AD from 1997 to 2021, have been examined [65].

## 3 MELATONIN RESTORES THE CIRCADIAN EQUIPOISE IN AD

### 3.1 The Cell Cycle-circadian Rhythms Interplay

Considering the underlying molecular mechanisms, circadian rhythms are conserved from drosophila to humans. The clock regulation of the cell cycle in the mammalian system is a multifactorial process, and any disruption in this system leads to changes in cell cycle characteristics. The functional clock components ubiquitously regulate optimal cell growth. Hence, any internal de-synchronization of the cerebral clocks results in pathophysiological alterations observed in many neurodegenerative disorders [66] where the aetiology and pathogenesis of AD and disrupted circadian rhythm share common factors [67]. First proposed to influence the timing of the cell cycle [68], the circadian clock later came to be known as a gating factor [69], the clock-dependent regulation of the cell cycle being an essential control mechanism. The reciprocal influence of the circadian clock and the cell cycle on each other suggests that these intertwined biological circuits are essential to ensure proper time-dependent mechanisms [70]. A few examples of such common regulatory proteins are mammalian Timeless protein (TIM), the mammalian silent mating type information regulation 2 homolog 1 (SIRT1), Period (Per) and Cryptochrome (Cry) that are all involved in the modulation of both circadian and cell cycles with overlapping functions. The major ‘clock’ genes include the period genes, Per1 and Per2, the cryptochrome genes, Cry1 and Cry2, the clock (circadian locomotor output cycles kaput) gene, and the Bmal1 (aryl hydrocarbon receptor nuclear translocator-like) gene. Clock and Bmal1 heterodimers act on E-box components of the promoters of the Per and Cry genes to stimulate transcription [71].

### 3.2 Melatonin’s Impact on Circadian Rhythms

**Fig. (1):**
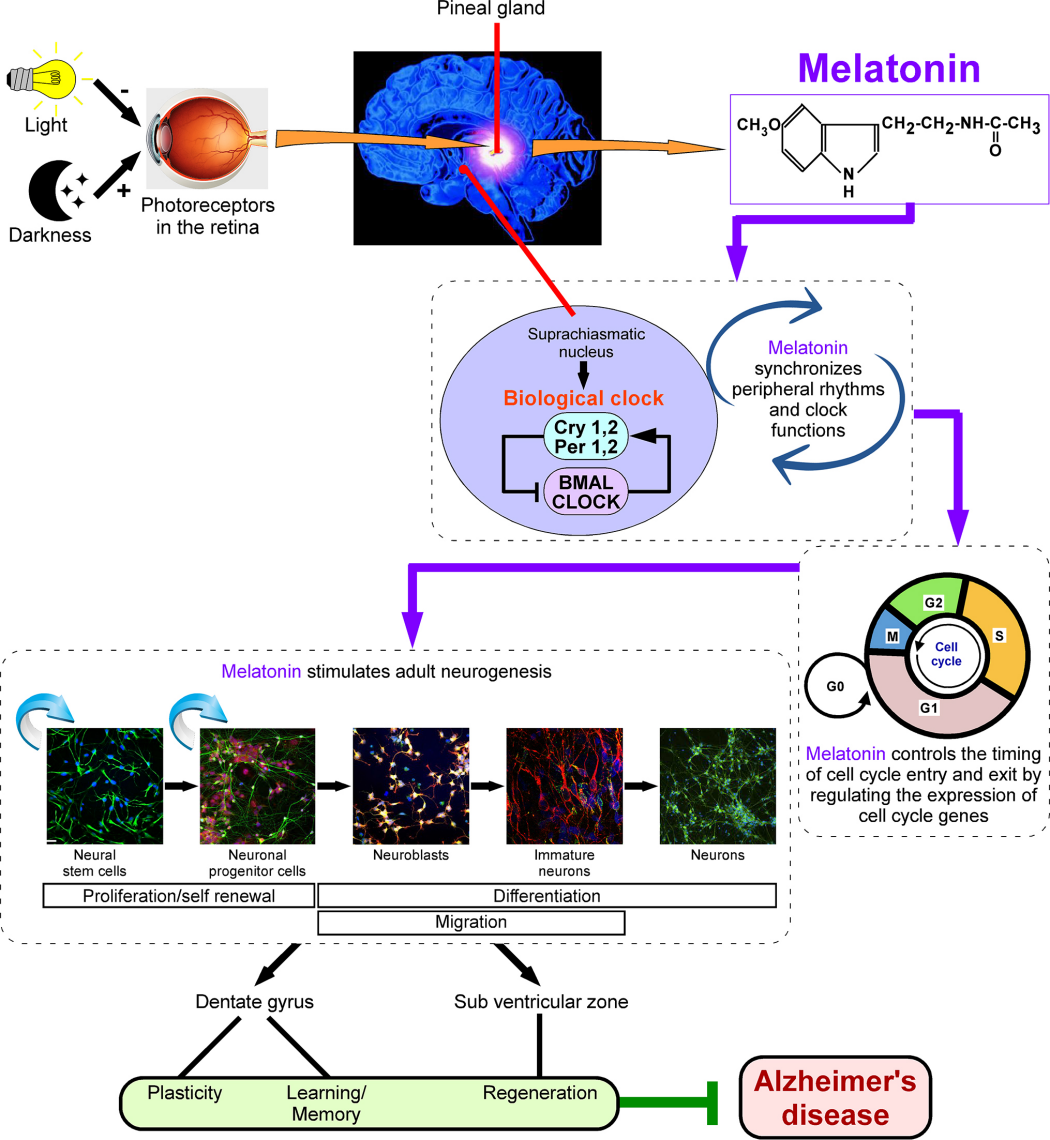
Role of melatonin in regulating circadian synchronization, cell cycle machinery and neurogenesis. The zeitgeber-like light synchronizes the daily rhythms that are generated in the suprachiasmatic nucleus (SCN) located in the hypothalamus. Melatonin plays a major role in peripheral clock synchronization by controlling clock genes and circadian rhythm. On the one hand, the molecular clock in the melatonin-producing cells of the pineal gland plays a key role in regulating the expression of cell cycle regulator genes, including cyclins, CDKs, CKIs, as well as checkpoint protein encoding genes, thereby preventing cell cycle re-entry-mediated apoptosis and degeneration. On the other hand, melatonin regulates time-dependent diurnal processes of cell proliferation and differentiation by augmenting the levels of stem cell markers, thus maintaining a homeostatic balance in the adult neurogenic niches.

Melatonin levels decline with advancing age, and aging alters the properties of the core transcriptional clock, as evidenced in flies [72]. Recently, it has been observed that loss of rhythmic Klf4 expression, a crucial transcription factor, in aged macrophages is associated with disruption of circadian innate immune homeostasis, a mechanism that may underlie the age-associated loss of protective immune responses [73]. In this context, melatonin promotes the stabilization of core pluripotency factors, such as Nanog, Sox2, Klf4, and c-Myc, by preventing m6A-dependent mRNA decay [74]. This draws particular attention to the age-associated decline of melatonin levels, which indicates the aging-associated disruption of circadian gene regulation and function. In addition, not only is this hormone a vital tool in the elderly, but inhibition of maternal melatonin also changes the expression of brain and muscle Arnt-like protein-1(Bmal1), Per2 and melatonin type-1 receptor in the foetal SCN [75]. These studies suggested that melatonin controls certain clock genes along with circadian rhythm (Fig. **1**). As such, the change in expression patterns of clock genes marks the rate of the aging process, and regulation of this phenomenon by melatonin signalling is yet another mechanism of how this indoleamine controls age-associated rhythmic processing.

### 3.3 Melatonin Alleviates AD-associated Circadian Disruption

It has been well recognized in rodent models that learning and memory have defined circadian variation throughout the day. Recently, circadian learning and memory performance has been evaluated using the single cosinor-based method in AD mice [76]. It has also been demonstrated in the 5XFAD model of AD that constitutive deletion of Rev-erbα (a circadian clock component and known as heme-responsive nuclear receptor protein) decreased amyloid plaque number and size and prevented plaque-associated amplification of disease-associated microglia markers, including Triggering Receptor Expressed On Myeloid Cells 2 (TREM2), lymphocyte common antigen (CD45) and C-Type Lectin Domain Containing 7A (Clec7a) [77]. Melatonin’s interaction with the proteasome and its involvement in the feedback loops (CRY/PER and REV-ERBα) [36] suggests another mechanism of how melatonin could possibly control Aβ clearance and neuroinflammation thus providing new insights into the role of circadian machinery in AD. Moreover, it has been previously shown that melatonin, at micro molar concentrations, potentiates the inhibitory effect of REV-ERB ligand SR9009 (which increases the constitutive repression of genes regulated by Rev-ErbA) in LX2 cells [78]. Although more exploration is needed to fully understand the relationship between melatonin and REV-ERBs in the context of neurodegeneration, significant disruptions of these interactions lead to altered memory performance and learning abilities, thereby suggesting that, by regulating circadian rhythms, melatonin could modulate learning and memory.

Circadian rhythm disruptions are more pronounced in neurodegenerative diseases like AD and related dementias, where they occur before the onset of typical clinical symptoms of neurodegeneration [15, 79]. Recently, the presence of circadian alterations and differences in long-term spatial memory, new object recognition memory and LTP, along with less robust locomotor activity rhythm, has been demonstrated in APP/PS1 mice [80]. During the early development of AD, there is a disruption of the normal expression of genes regulating circadian function after exposure to light, particularly in the SCN but also in extra-hypothalamic brain areas supporting circadian regulation, suggesting a severe impairment of the functioning of the clock gene pathway as investigated in a triple transgenic model of AD (3×Tg-AD) and their wild type littermates [81]. Importantly, the circadian redox regulation of neuronal excitability extends from the SCN to the hippocampus, which gives insights to understand the hippocampal circadian processes, such as learning and memory, along the course of aging and neurodegeneration [82]. It has also been observed that epigenetic alterations in the SCN and hippocampus elicit cognitive decline and memory impairments in rodents [83], while a study in an APP/PS1 transgenic mouse model of AD has evidenced that circadian disturbances might occur early during the stage of AD pathogenesis [80]. As such, an altered synchronization of circadian rhythmicity in the brain of AD patients has further reinforced the idea that circadian oscillators are key factors in the development of this devastating disorder [84, 85]. Recent clinical evaluation of the regional distribution of Aβ in the brain by positron emission tomography-computed tomography (PET-CT) standardized uptake value ratios (SUVRs) in normal healthy, MCI and AD dementia groups showed that in AD patients, the alterations in circadian rhythms are well associated with amyloid burden [86], which provides a clear-cut indication of how alterations in circadian rhythm result in neurodegeneration due to Aβ deposition. Importantly, this phenomenon runs parallel with the declining levels of melatonin observed in AD. Moreover, the deregulated diurnal variation in clock gene expression in the hippocampus corresponds with a loss of normal day/night differences in membrane excitability, synaptic physiology and cognition in APP transgenic mice [87]. As melatonin modulates circadian rhythms and regulates the Aβ biology in terms of production, conformational changes, oligomerization, fibrillation, and ultimately senile plaque formation [88], its therapeutic implementation seems legitimate [89].

Interestingly, tau function is also essential for the regulation of the circadian network and associated behaviours. Indeed, alterations in tau homeostasis deregulate the structural plasticity of the ventral lateral circadian pacemaker neurons by disrupting the temporal cytoskeletal remodelling of its dorsal axonal projections and by inducing a slight increase in the cytoplasmic accumulation of core clock proteins [90]. In this particular context, it has been shown that melatonin treatment in mouse and human *ex vivo* and *in vivo* tau-related models provides some beneficial effects and prevents cognitive decline [91]. Additionally, melatonin regulates tau phosphorylation by affecting the function of kinases and phosphatases [92] and reduces the aggregation propensity of tau by inhibiting its aggregation and dissolving the preformed aggregates [93].

Because altered circadian clocks act as a risk factor for the development of neurodegenerative diseases *via* an altered production or clearance rates of toxic metabolites such as Aβ, they could be potential therapeutic targets for attenuating the onsets and progressions of these disorders [94]. Supporting this possibility, a randomized clinical trial has revealed that interventions targeting the circadian system improve sleep, mood and behaviour in patients with AD and related dementias [95]. Since not only dysfunction in clock gene but also altered melatonin secretion has been reported in AD and other neurodegenerative diseases [96] and considering the interrelationship between the circadian clock and neurodegeneration, the prospects of using melatonin is of prime interest in the context of circadian disruption being an emerging link to the aetiology of AD [97]. This hypothesis is supported by the fact that melatonin has indeed been reported to regulate aging and neurodegeneration through circadian rhythm pathways [98].

## 4 THE CIRCADIAN RHYTHM/CELL CYCLE/MELATONIN CONNEXION

Several mammalian cell cycle genes, such as *c-Myc, cyclin D1* and *Wee-*1, are regulated in a circadian fashion [99] and mutations in various clock genes have been shown to result in the modulation of several cell cycle-associated genes [100]. As an example, Wee1 expression is controlled by the clock gene complex and contributes to cell-cycle progression. Wee1 is a critical coordinator of the transition between DNA replication and mitosis and yet another mitotic regulator that participates in the AD neurodegenerative process [101]. Therefore, it could be speculated that an aberrant expression of circadian clock genes can lead to atypical expression of their downstream targets that are involved in cell proliferation and apoptosis. As melatonin is itself a circadian regulator, it could possibly affect the downstream effectors of the circadian pathway both in physiological and pathological conditions.

Importantly, the formation of the embryonic brain requires the production, migration, and differentiation of neurons to be timely and coordinated with both light and melatonin scheduling the differentiation of neurons and the formation of neural processes as seen in the habenular nuclei of zebra fish [102]. Interestingly, neurogenesis in the adult hippocampus occurs in a time-of-day dependent fashion, which may dictate daily modifications of dentate gyrus physiology. The hippocampus is subjected to diurnal/circadian rhythms on both the morphological and molecular levels. A study in the animal model showed that melatonin receptor type 1 and 2 (MT1/2)-mediated signaling appears to be crucial for the generation and timing of zeitgeber time-dependent changes in cell proliferation and apoptosis and for differentiation of proliferating cells into neurons in the sub granular zone (SGZ) [103].

Daily variations of neural progenitor divisions and neurogenesis in the adult mouse brain have been demonstrated. During night time, the progenitors actively enter M-phase, thereby giving rise to more neuronal progeny [104]. The possibility that light-controlled rhythms are a primary regulator of neuronal proliferation exemplifies the complexity of the circadian control pathway of the neuronal cell cycle [105]. Nevertheless, it has been shown in neurogenic niches of an adult diurnal vertebrate that the circadian modulation of cell cycle progression involves both systemic and niche-specific factors [106], implying that the cell cycle progression displays a robust circadian pattern. Another study in the dentate gyrus of knock-out mouse models provides insight into how cell autonomous circadian clocks and clock genes regulate adult neural stem cells with implications for treating neurodegenerative disorders and impaired brain functions by manipulating neurogenesis [107]. Therefore, understanding the circadian regulation of cell cycle machinery can help optimize the timing of therapeutic approaches in patients with neurodegenerative disorders.

As the circadian molecular clock regulates adult hippocampal neurogenesis by controlling the timing of cell-cycle entry and exit [108], it is of utmost importance to investigate the underlying mechanisms of this regulation and the functional role of melatonin as this neurohormone acts as both circadian and cell cycle regulator (Fig. **1**). This is supported by the timing of gene expression for critical cell cycle regulators cyclins D, A2, and B2 and cyclin-dependent kinase inhibitor p20 in brain tissue [106]. Also, cellular senescence is characterized by the altered expression of cell-cycle proteins, particularly the up-regulation of cyclin-dependent kinase inhibitors such as p16 and p21 [109]. Because the molecular clock in the melatonin-producing cells of the pineal gland plays a key role in modulating circadian behavior [110], since circadian clocks are considered potential therapeutic targets to attenuate onsets and progressions of neurodegenerative diseases like AD [94], and considering melatonin therapy as an advantageous modality in terms of therapeutics [111], using melatonin-based approaches to mitigate circadian rhythm disruptions in aging and neurodegenerative diseases would certainly delay the onset or abate the development of AD pathogenesis.

## 5 MELATONIN MITIGATES THE AD-DEPENDENT DEREGULATION OF THE CELL CYCLE MACHINERY

### 5.1 The Cell Cycle Balance is Disrupted in AD

Neurogenesis involves proliferation and differentiation, which implies, in cell cycle terms, re-entering and exiting the cell cycle, these two processes being interlinked [112]. The neuronal fates are usually determined around their final cell cycle [113-115], and the proteins that drive or inhibit the cell cycle include cyclins, cyclin-dependent kinases (Cdks), cyclin-dependent kinase inhibitors (CKIs), DNA replication proteins and checkpoint proteins [116].

The cell cycle regulators in adult neurons are involved in functions such as neuronal migration, neuronal maturation and synaptic plasticity [117, 118], and their enhanced expression in response to toxic insults and oxidative stress [119] triggers a number of signalling pathways in various diseases [120] including AD [121]. Importantly, neurons die in AD because of erroneous cell cycle control before the pathological hallmarks appear [122]. Thus, the up regulation of the cell cycle proteins cyclin D1, cyclin E, cyclin A, Cdk2, and E2F transcription factor 1 (E2F-1), along with increased phosphorylation of the retinoblastoma tumour suppressor gene (Rb) protein, results in aberrant cell cycle re-entry and subsequent apoptosis [119], thereby contributing to AD progression [123], in which the cells are prone to dedifferentiation and degeneration [124]. Therefore, therapeutic interventions aimed at ameliorating mitotic changes is expected to have a profound impact on AD progression [125, 126].

Recently, an integrative functional genomic analysis was performed to identify pivotal regulators of the cell cycle and their underlying pathways for developing optimal treatment of AD. This study revealed 775 cell cycle genes and 77 trophic factor receptors differentially expressed in AD when compared to controls [127]. Additionally, a recent combined meta-analysis and co-expression analysis identified 18, 2061 differentially expressed genes and 36 differentially expressed miRNAs, including genes involved in the neuronal cell cycle re-entry, the ubiquitination/proteasome system and mitochondrial homeostasis as well as miRNAs associated with AD pathology. Among them, UBC, ESR1, EGFR, CUL3 and KRAS genes were identified as cell cycle re-entry related factors, thereby suggesting involvement of cellular dyshomeostasis and receptors mediating Aβ toxicity from impaired ubiquitination proteasome system in the cell cycle re-entry occurring in AD [128].

Hippocampal neurons in mild cognitive impairment (MCI) patients express proliferating cell nuclear antigen (PCNA), cyclin D and cyclin B [15], and CKIs such as p16 (INK4a), p18 (INK4c) and p27 (KIP1) are expressed in neurons of AD patients [129, 130], indicating the integral involvement of the cell cycle machinery in affected neurons. The regulation of cell cycle by different cyclins and CDKs is well counter balanced by their inhibitors, such as p27(Kip1) that has the ability to control the different phases of the cell cycle at the nuclear level. Importantly, alterations in the levels and phosphorylation status of p27(Kip1) have been observed in AD with excessive Aβ_42_ production, tau hyper phosphorylation and deregulated insulin signalling leading to an imbalance between the levels and functions of p27(Kip1) in the cytoplasm and nucleus by causing alterations in p27(Kip1) post-transcriptional modifications, which eventually leads to an aberrant cell cycle re-entry [131]. In this context, inhibition of constitutively active NF-κB *via* melatonin MT1 receptor can de-repress the p27(Kip1) promoter leading to transcriptional up-regulation of p27(Kip1), which has been demonstrated in cancerous cells [132], whether a similar interaction of melatonin with p27(Kip1) occurs in neuronal cells certainly deserves further investigation. Moreover, Aβ peptides reactivate the cell cycle machinery of neurons and cause aberrant activation of mitogen-activated protein kinase/extracellular signal-regulated kinase (MEK/ERK) signalling, which promotes the entry of neurons into the cell cycle, ultimately resulting in apoptosis [133] by elevating the levels of cyclin D1 [134]. Finally, Cdk4 is activated in the AD brain and its inhibition ameliorates the neurodegenerative process in AD [135].

### 5.2 Melatonin Promotes Cell Cycle Homeostasis Recovery

#### 5.2.1 p75NTR

The anti-apoptotic effects of melatonin are mediated by two potential mechanisms: by increasing the activity of pro survival pathways *via* protein kinase B (Akt) and by the prevention of DNA damage followed by the reduction of cell cycle re-entry [136]. Cell cycle re-entry in AD may be regulated by microRNA (miR)-26b since the levels of this miR are elevated in relevant pathological areas from the early stages of the disease [137]. Melatonin has been shown beneficial in alleviating many aspects of AD *via* regulating some miRs [35], but its link with miR-26b needs further exploration. The p75 neurotrophin receptor (p75NTR) has emerged as a potential target for the regulation of neural plasticity and the promotion of adult neurogenesis in response to injury and neurodegenerative diseases. Thus, it is up regulated under pathological conditions and accounts for controlling numerous processes necessary for nervous system recovery [138]. As p75NTR is involved in the production of Aβ, neuronal death, neurite degeneration, tau hyper phosphorylation, cell cycle re-entry and cognition decline in AD [139], it is considered a novel therapeutic target and as a biomarker for AD [140] and also aids in increasing survival signalling in neurons [141]. In this context, melatonin alleviates Aβ-induced alterations in tropomyosin receptor kinase A (TrkA) and p75NTR protein levels in SHSY5Y neuroblastoma cell cultures [142, 143].

#### 5.2.2 Cyclins and Cdks

Cell cycle markers that are normally found in the nucleus, where they are needed for the expression of key genes that allow the diseased neuron to continue to G1 and, eventually, apoptosis, are elevated in AD neurons [121]. Moreover, treatment of cells with Aβ peptide was shown to relocate Cdk5, which controls the cell cycle in mature neurons, from the nucleus to the cytoplasm, with a paralleled cell cycle re-entry [144]. This type of Aβ-induced cell cycle re-entry was shown to immediately precede neuronal cell death in AD [145]. It has been demonstrated that melatonin attenuates 1-methyl-4-phenyl-1, 2, 3, 6-tetrahydropyridine (MPTP)-induced neurotoxicity *via* preventing Cdk5-mediated autophagy in the brains of monkey and mice [146], which implies an effective regulation of Cdks by this indoleamine.

Cdk5 and its neuron-specific activator p35 are required for neurites outgrowth and cortical lamination and the cleavage of p35 to p25 is considered to be one of the reasons for Cdk5 deregulation and hyper activation in AD [147] as the hyperactivated p25/Cdk5 complex both enhances β-secretase (BACE1) activity *via* phosphorylation [148] and hyper phosphorylates tau [149], thereby disrupting the cytoskeleton and promoting the apoptosis of neurons. Thus, p25/Cdk5 is impacting both amyloid and tau pathologies in AD [150]. Furthermore, Cdk5 is important for cell cycle arrest of post mitotic neurons [144], and Aβ_42_ alters the localization of Cdk5 and promotes neuronal cell cycle re-entry [151, 152]. Moreover, the inhibition of this kinase provides neuroprotection against AD [153]. Related to this, melatonin has been shown to prevent tau hyper phosphorylation by reducing Cdk5 expression as well as the cleavage of p35 to p25 [154-156]. In addition, there exists a molecular link between melatonin and its effects on the cell cycle [157] and on cell cycle markers such as cyclin D1, B1, Cdk1 and Cdk4 [136, 157, 158]. Finally, cleavage of p35 to p25 by the calcium-dependent cysteine protease calpain may be involved in the pathogenesis of AD, as suggested by the fact that specific inhibitors of this enzyme effectively inhibit the calcium-induced cleavage of p35 and Aβ-induced cell death [159]. Importantly, melatonin substantially prevents the activation of calpain and caspase-3 and ameliorates dexamethasone-induced neurotoxicity in human neuroblastoma cells [160].

The Cdk inhibitor p16INK4a is elevated in AD and is associated with neurofibrillary degeneration [129], as well as other members of the INK4-family of cyclin-dependent kinase inhibitors such as p15INK4b, p18INK4c and p19INK4d that bind directly to Cdk4/6 or to complexes of Cdk4/6 with D-type cyclins [130]. Cdk4 is activated in the AD brain, and its inhibition is considered a therapeutic track for ameliorating neurodegenerative processes in AD [142]. In this regard, melatonin has been shown to down regulate Cdk4 in osteoblastic cell cultures [161], implying its regulation over such kinases. Moreover, elevated glucocorticoid levels suppress hippocampal neurogenesis in adults where G1 cell cycle arrest is mediated by an increase of cyclin/cyclin-dependent kinase inhibitor p21, and melatonin prevents glucocorticoids-mediated toxicity and inhibition of cell proliferation in hippocampal cells of the rat brain [162].

#### 5.2.3 DYRK1A/E2F-1/NFATs

Dual-specificity tyrosine phosphorylation-regulated kinase 1A (DYRK1A) is a protein kinase with diverse functions in neuronal development and adult brain physiology, and its enhanced levels are associated with the pathology of neurodegenerative diseases like AD [163]. DYRK1A is implicated in Aβ-mediated tau hyper phosphorylation [164], and its inhibition has come out as a potential treatment for AD [165]. This kinase controls cyclin D1 and p21 protein levels, and the p21-cyclin D1 signalling map specifies cell cycle entry and exit decisions [166]. Of note, Aβ peptides induce a significant increase in DYRK1A mRNA in neuroblastoma cell cultures [167]. Regarding the mechanisms of action, it is known that the transcription factor targets of retinoblastoma protein (E2F-1) increases DYRK1A mRNA levels by enhancing its promoter activity [168]. E2F-1 in the AD brain is found primarily in the cytoplasm rather than its normal location in the nucleus, where it normally regulates neuronal cell death induced by DNA damage, thus suggesting that the liberation of E2F-1 from the nucleus exacerbates apoptosis in the AD brain [169]. Melatonin reverses Fas, E2F-1 and endoplasmic reticulum stress-mediated apoptosis and deregulation of autophagy in mice model [170]. The nuclear factor of activated T-cells (NFATs) is also involved in regulating DYRK1A levels [171, 172]. The fact that melatonin mediates its anti-apoptotic effects by increasing the activity of prosurvival pathways by the prevention of DNA damage by regulating E2F-1, followed by the reduction of cell cycle re-entry [136], along with its involvement in regulating NFATs [173], gives an insight about how melatonin could possibly regulate the levels of DYRK1A and its functioning.

#### 5.2.4 FoxG1

Related to the possible targeting of cell cycle re-entry as a therapeutic intervention for AD, a very recent investigation has reported involvement of Forkhead Box G1 (FoxG1), a transcription factor which plays an important role in brain development in AD. Using the APP/PS1-Foxg1fl/fl-CreAAV mouse line, it was shown that FoxG1 negatively regulates the levels of p21-activated kinase (PAK3) and likely antagonizes cell cycle re-entry with FoxG1 blocking neuronal apoptosis and Aβ deposition *via* the alteration of p21cip1-mediated arrest at the G2 stage and the regulation of cyclin A1- and cyclin B-dependent progression of the cell cycle [174]. This altogether clearly indicates that a pharmacological augmentation of FoxG1 levels could indeed represent a therapeutic track for AD. Although little is known about the interaction between melatonin and FoxG1, developmental studies have shown that FoxG1 mutants exhibit a reduced pineal gland [175]. Thorough investigations will be required in the future to draw any inference from the aforementioned link.

#### 5.2.5 Mcm/PCNA

Aberrant reactivation of cell-cycle mechanisms is also a feature of the aging brain. The mini chromosome maintenance proteins (Mcms) are a eukaryotic family of six distinct protein subtypes (Mcm2-7) that are necessary for DNA replication initiation and progression in the cell cycle [176]. Mcm2 is a marker for chromosomal replication licensing, which is increased in the hippocampus of elderly brains with AD type pathology along with increased expression of Ki67 and Proliferating cell nuclear antigen (PCNA) [177]. Furthermore, phosphorylated Mcm2 is markedly associated with neurofibrillary tangles, neuropil threads, and dystrophic neurites in AD [178]. Cell cycle events play a major role in the loss of neurons not only in the advanced stages of AD but are also present in MCI individuals, as shown by the fact that PCNA, cyclin D, cyclin B and Cdk4 expressions are increased in the hippocampus and in the basal nucleus of Meynert from MCI individuals similar to that of AD patients [20, 179]. Although melatonin, *via* its receptor activation, down regulates the protein expression of PCNA in rat chondrocytes [180], delineating its detailed interaction with Mcms needs further exploration.

#### 5.2.6 ATM/p53

Neuronal cell cycle checkpoint proteins, together with the DNA damage response genes Mouse double minute 4 homolog (MDM4), Ataxia-telangiectasia mutated (ATM) and ATM- and Rad3-Related (ATR), are strongly up regulated and associated with the progression of dementia. In addition, the downstream target of ATM-p53 signaling, TP53 induced glycolysis regulatory phosphatase (TIGAR), a p53-inducible protein, the activation of which can regulate energy metabolism and protect against oxidative stress, is progressively decreased as the severity of dementia evolved [181]. Interestingly, melatonin significantly prevents DNA damage *via* ATM inhibition [182] and regulates DNA damage response at the transduction, mediation as well as functional levels [183].

p53, an important factor involved in many areas of cellular physiology and biology, ranging from cellular development and differentiation to cell cycle arrest and apoptosis, along with important functions related to neurodegeneration and synaptic plasticity, is highly elevated in AD [184, 185] and induces tau phosphorylation [185]. Considering that the neuronal cell cycle re-entry coincides with tau hyper phosphorylation and precedes cell death [186], melatonin, along with preventing the hyper phosphorylation of tau, also protects against apoptosis by regulating p53 levels in rat cerebrum [187].

#### 5.2.7 Autophagy/FOXO/Cdk1

Autophagy-related gene expression is regulated by forkhead transcription factor (FOXO)/a NAD-dependent histone deacetylase (SIR2)-mediated aging process, which in turn is associated with the onset of AD [188]. Given that the regulation of FOXO3a contributes to prevent AD-like amyloid pathology [189], melatonin increases the expression of SIRT1 and FOXO3a in senescence-accelerated mice [190] and regulates hippocampal neuronal homeostasis by increasing SIRT1, FOXO1 and melatonin receptors expression in the aging hippocampus [191]. Cdk1 has been shown to be an apoptotic kinase by inducing the phosphorylation of FOXO1 at the G2/M checkpoint leading to the expression of Polo-like kinase 1 (Plk1), a key regulator in bridging the assembly and dynamics of the spindle fibres to regulate cyclin/CDK complexes [192]. Interestingly, the inhibition of Cdk1 provides neuroprotection through the blockade of aberrant cell cycle progression [193], while Plk1 activity is elevated in the AD patient brain [194]. Conversely, inhibition of Plk1 kinase activity or depletion of Plk1 reduces Aβ-induced neuronal cell death [195]. Directly related to this, melatonin significantly inhibits cell proliferation in a dose-dependent and time-dependent manner, and this inhibition involves the down regulation of cyclin D1, Cdk4, cyclin B1 and Cdk1 [158] by inhibition of the ERK1/2 signalling [196]. As a reminder, ERK activity regulates the induction of cyclin D1 [197], and the Aβ_1-42_ peptide causes aberrant activation of MEK/ERK signalling, which promotes the entry of neurons into the cell cycle, thereby resulting in their apoptosis [198].

## 6 IMPLICATIONS OF βAPP AND TAU BIOLOGY IN THE MELATONIN-DEPENDENT STABILIZATION OF THE CELL CYCLE DYNAMIC IN AD

Cell cycle-dependent changes can regulate βAPP metabolism and tau hyperphosphorylation, while, in return, tau mutation triggers an abnormal cytoplasmic localization of cell cycle-associated factors [199-201]. Next to this, the inhibition of melatonin biosynthesis not only impairs spatial memory in animal models but also increases tau phosphorylation and activates glycogen synthase kinase-3 beta (GSK3β) [202], while its supplementation attenuates tau hyper phosphorylation [203]. Last but not least, neuronal cell cycle events also occur in the absence of Aβ deposition and depend upon the amyloidogenic processing of βAPP [204] as high levels of Aβ peptides induce apoptosis, while they induce cell cycle re-entry in neurons at low levels [145, 198]. In this general context, melatonin has been largely established as a protective factor against the amyloid-dependent development of AD [88] and has been more particularly shown to regulate the non-amyloidogenic and amyloidogenic processing of βAPP under both physiological [205, 206] and pathological [207] conditions, which suggests that a reduction in the physiological level of melatonin might induce these Aβ-dependent changes much before the aggregation occurs.

### 6.1 Presenilin/p53/p21/Ras

The P117R mutation on presenilin 1, the catalytic core of the βAPP-cleaving γ-secretase, increases the levels of p53 and p21, and these changes are rescued by p53 inhibition [208]. Also, the cancer-associated variant of p21^cip1^ may contribute to the loss of cell cycle control in neurons, potentially leading to AD-like neurodegeneration [209]. In this context, melatonin has been shown to decrease p53 activation *via* an up regulation of SIRT1 [210] and to reduce the number of p21-positive cells in the SGZ of the dentate gyrus in adult rats [211]. Additionally, melatonin treatment also attenuates the expression of p21 and p53 in ligament stem cells [212]. Ras proteins play an essential role in the transduction of mitogenic signals from activated growth-factor receptors, leading to the cell-cycle entry. The early p21Ras expression pathway is activated during the post-translational modification of βAPP and the phosphorylation of tau, which might also involve PCNA and Ki-67 [213]. As melatonin functions to inhibit Ras [214], it can be speculated that this action of melatonin might also be involved in the regulation of cell cycle-mediated processes. Such links clearly indicate an intense association between cell cycle-related proteins and cardinal hallmarks of AD, where melatonin plays a pivotal role.

### 6.2 APP-BP1/NEDD8

Moreover, the fact that melatonin modulates caspase activity [160], thereby preventing apoptosis [215, 216], suggests that the hormone could lessen the cell cycle re-entry-induced and caspase-dependent apoptosis taking place in AD [22], while the ability of melatonin to inhibit the ubiquitination-dependent proteasome pathway may provide a promising track in the quest to treat neurodegenerative disorders like AD [217] when considering that the ubiquitin-proteasome system (UPS) is involved in the cell cycle progression and is deregulated in AD [218]. Moreover, APP-BP1, a βAPP-binding protein, is the regulatory subunit of the activating enzyme for the small ubiquitin-like protein, neural precursor cell expressed developmentally down-regulated protein 8 (NEDD8) [219], and the interaction of APP-BP1 with βAPP plays a role in cell cycle transit control [220]. Neddylation is indeed a post-translational modification that controls ubiquitination and the NEDD8 system is essential for the regulation of protein degradation pathways involved in cell cycle progression [221]. Given that deregulated neddylation has been established as a feature of AD pathogenesis [222], it has been demonstrated that melatonin pre-treatment prevents the Aβ_42_-induced enhancement of APP-BP1 protein expression and the alteration of NEDD8 cellular localization [223].

### 6.3 Oxidative Stress/BACE1

The high rate of oxidative metabolism and low levels of endogenous antioxidant enzymes in the brain marks the susceptibility and sensitivity of neurons towards ROS/reactive nitrogen species (RNS) levels, and oxidative stress may cause DNA damage affecting the cell cycle progression and altering the genomic integrity as it stimulates quiescent neurons to leave the G0 phase and re-enter the cell cycle [224]. Re-entry into the cell cycle in terminally differentiated neurons influences cell senescence and further contributes to oxidative stress and deregulated apoptosis [225], which is a prevalent feature in AD [226]. Likewise, under conditions of increased oxidative toxicity, AD neurons possess genetic defects allowing them to enter and stay in the G1 phase of the cell cycle instead of proceeding towards apoptosis.

Moreover, even a mild form of oxidative stress promotes the amyloidogenic processing of βAPP by redistributing BACE1 [227] and increasing its protein levels [228]. Interestingly, the neurofibrillary tangle-bearing neurons overexpress more than two hundred genes involved in oxidative stress [229]. Age-associated decrease in melatonin levels [44] and its parallel decrement with AD progression [230] makes the brain more vulnerable to oxidative damage-induced cell cycle homeostasis disruption. Relatively, melatonin enhances the expression of a number of antioxidant enzymes that are reduced in AD [231], not only in the brain but also in various other organs [232].

In addition, melatonin-induced reduction in tangle formation would also be beneficial in preventing the instigation of overexpression of genes pertaining to oxidative stress [210]. Neurons are vulnerable to ROS-induced DNA damage that correlates with cell cycle arrest [233], and melatonin significantly attenuates DNA damage in many experimental models [234]. Silent information regulator 2 (SIR2) activity is dependent on its cofactor nicotinamide adenine dinucleotide (NAD+) and decreases in NAD+ levels occurring due to the disruption of metabolic processes, would limit SIR2 activity and trigger cell cycle re-entry [235]. Also, NAD+ has been shown to be beneficial in AD by regulating BACE1 levels and reducing Aβ toxicity [236]. Related to this, melatonin is a potent free radical scavenger [237] and acts as an effective antioxidant [238] to preserve NAD+ levels under oxidative stress [239], which suggests that melatonin prevents oxidative stress-induced aberrations in cell cycle events not only because of its intrinsic antioxidant properties but also by boosting the endogenous levels of brain antioxidant enzymes [240].

Table **1** summarizes the beneficial effects of melatonin on cell cycle-related factors.

## 7 ROLE OF MELATONIN IN REGULATING THE CELL CYCLE DURING NEUROGENESIS

Cell cycle kinetics modulates neurogenesis, and it is the cell cycle length that plays an alleged role in defective neurogenesis [241]. The cell cycle machinery is involved in the regulation of quiescence and expansion of stem cells and in the differentiation of neural progenitors in the adult neurogenic niches [242-244], along with the regulation of histogenesis, neuronal differentiation and maintenance of stem cells [112]. The cell cycle regulators directly influence mechanisms that control the cell fate and differentiation in the developing nervous system, thereby playing important roles in controlling cell cycle progression and neurogenesis [245].

Adult neural stem cells (NSCs) are heterogeneous [246] and can be affected by differences in cell signalling [247], and changes in cell cycle parameters and re-entry of adult neural or stem cells reflect cell cycle variation in pathological versus physiological conditions [248]. Thus, the complex balance between neurogenerative and neurodegenerative processes functions relies on the fine-tuning of programmed cell death, which goes awry in neurodegeneration and leads to untimely and unnecessary loss of neurons. The regulation of cell cycle entry and exit is crucial for developing neurons, and the transcription factors paired box (Pax) and SRY (sex determining region Y) box (SOX) family of proteins regulate and crosstalk with cell cycle regulatory proteins [249]. Importantly, melatonin has been shown to increase Sox2 expression [250, 251], thereby supposedly preventing abnormal cell cycle re-entry and maintenance of stemness (Fig. **1**). Interestingly, homeobox genes, that are required for normal development of the rodent pineal gland, include *Pax6* [252], which expression is concomitant with the ontogenetic onset of melatonin synthesis [253].

Beta-catenin signaling is an important modulator of both cell cycle mechanics and apoptosis [254] and interruption of this signaling may contribute to the impairment of progenitor cells' neurogenesis in AD [255]. Melatonin activates β-catenin signaling and protects neurons against prion protein-induced neurotoxicity [256], and where cell cycle regulatory proteins may affect the proliferative activity of NPCs across the lifespan, which affects the rate of cell division [257], melatonin treatment prevents the alteration of several markers involved in cell cycle progression and synaptogenesis in NSCs derived from the adult rat hippocampus [258].

The age-related decline in neurogenesis has been attributed to a decreased pool of NPCs because aging cells divide less at a given period, and the ones which do are more likely to re-enter the cell cycle within a day, both *in vitro* and *in vivo* [259]. Although mesenchymal stem cells represent an attractive source for stem cell-based regenerative therapy [260], they are vulnerable to oxidative stress-induced premature senescence under pathological conditions. In this context, melatonin interestingly protects these cells from premature senescence *via* the SIRT1-dependent pathway by improving cell proliferation, decreasing senescence-associated β-galactosidase activity, and improving entry of proliferating cells into the S phase by attenuating the phosphorylation of p38 mitogen-activated protein kinase (MAPK), decreasing the expression of the senescence-associated protein p16 (INK) (4α), and increasing SIRT1 [261].

G protein-coupled receptors are additional key regulators of cellular communication that can regulate the expression, activity, localization and stability of cell cycle regulatory proteins that either promote or inhibit the initiation of DNA synthesis. These receptors regulate cellular proliferation, specifically the progression from the G1 phase to the S phase of the cell cycle [262]. Both MT1 and MT2 melatonin receptors are G protein-coupled receptors that, apart from regulating a plethora of physiological processes, also regulate cell proliferation [263] as well as neurogenesis under pathophysiological conditions [37].

Moreover, different proteins associated with the cell cycle, including cyclins, cyclin-dependent kinases and proto-oncogenes such as c-Myc, are increased in degenerating neurons. The proto-oncogene Myc induces neurons to re-enter the cell cycle [264], which is pathophysiologically relevant to AD and other neurodegenerative diseases. Thus, such cell cycle re-entry in primary neurons leads to tau phosphorylation and conformational changes, as observed in AD [26], as well as neuronal cell death, gliosis and cognitive deficits. In direct connexion with these events, while melatonin drives the circadian amplitudes of proliferation genes like c-Myc [265, 266], it also reduces c-Myc expression [267] to potentially prevent AD-associated excessive cell cycle re-entry. From a mechanistic point of view, c-Myc levels are positively controlled by ERK, which phosphorylates and stabilizes c-Myc and thus allows it to act on growth and transformation genes promoters [268], with aberrant activation of MEK/ERK signalling, promoting the entry of neurons into the cell cycle and their apoptosis [134]. Because melatonin down regulates ERK [63], a clear inference can be drawn that melatonin, directly or *via* the ERK signalling pathways, regulates c-Myc, which in turn could possibly be beneficial in preventing c-Myc-induced excessive cell cycle re-entry observed in AD.

Another key actor of neurogenesis is the Ki-67 transcription factor that is expressed in proliferating progenitors in S, G_2_, M, and parts of G_1_ phases of the cell cycle [269], and that is associated with AD-related cytoskeletal pathology [270]. Interestingly, melatonin significantly restores Ki67-positive proliferating cells in the dentate gyrus of the D-galactose-induced mouse model *via* an increase in cyclic AMP response element binding (pCREB) expression [271], which is suggestive of the fact that melatonin may be helpful in reducing age-related phenomena in the brain. In addition, melatonin-induced ERK activation regulates c-Myc protein stability and plays a principal role in controlling cell cycle progression and apoptosis [272].

Brain-derived neurotrophic factor (BDNF) stands as another important neurogenesis-promoting factor having an impact on the cell cycle by positively controlling the expression of cyclin D1 and cyclin E [273, 274]. Given that melatonin has been described as being able, alone [275, 276] or in combination with exercise [277], to induce an increase in BDNF brain levels in animals, one can suppose that melatonin acts in a stimulating way on neurogenesis partially *via* its ability to raise BDNF levels and thus promote NSCs proliferation. However, the lack of evidence for a stimulatory effect of melatonin on serum BDNF levels in humans invites caution regarding this hypothesis [278].

Table **2** presents a digest of the beneficial effects of melatonin on neurogenic factors.

Finally, because melatonin is a powerful chronobiotic able to potently regulate the sleep/wake cycle [54], one should consider its possible differential impact on neurogenesis during rapid eye movement (REM) and non REM sleep in an AD context. Indeed, REM sleep-associated processes facilitate the proliferation of granule cells in the adult hippocampal and its deprivation has been shown to reduce neurogenesis in the hippocampal dentate gyrus of the adult rat [279]. Recently, it has been demonstrated that REM sleep deprivation alters learning-induced cell proliferation and the generation of newborn young neurons in the dentate gyrus of the dorsal hippocampus [280]. In this context, a differential involvement of the melatonin receptors MT1 and MT2 in the regulation of REM and non-REM sleep is envisioned since MT_2_ receptors are located in the reticular thalamus (non-REM area), while MT_1_ receptors mostly reside in the locus coeruleus and the lateral hypothalamus (REM areas) [281]. Moreover, animal studies have also shown that a prophylactic administration of oral melatonin for two weeks in REM sleep-deprived mice increases the proliferation of neural progenitors and the levels of antiapoptotic proteins in the hippocampus [282]. Recent studies have suggested that clock-timed melatonin treatment can reduce the development of Parkinsonism and alleviate REM sleep behaviour disorder (RBD). Firstly, an observational cohort study demonstrated that low-dose, long-term and timely clocked melatonin treatment in RBD patients effectively improves RBD symptoms [283, 284]. Secondly, considering that RBD patients tend to develop α-synucleinopathy within a decade of RBD onset, daily bedtime administration of melatonin has been effective in reducing RDB, which further might halt PD-leading neurodegeneration [285]. However, when considering AD, there is to date no solid proof that RDB is directly linked to an increased probability of developing the disease, and a fortiori no data evidencing a beneficial effect of melatonin on the pathology *via* its action on REM [286].

Nevertheless, the supposed ability of melatonin to increase human adult neurogenesis *via* its ability to reduce sleep disorders is based on several observations. Firstly, it has been established that disruption in sleep for more than twenty-four hours substantially inhibits cell proliferation and decreases cell survival suggesting that sleep has a crucial role in regulating neurogenesis with subsequent effects on learning and memory [287, 288]. Secondly, REM sleep deprivation reduces hippocampal neurogenesis [279, 289] and hippocampal volume with significant alterations in neuronal plasticity and cognitive functions [290]. Thirdly, sleep reduces DNA damage and positively modulates chromosome dynamics [291]. In this context, DNA damage is associated with aging and age-related neurodegenerative disorders by significantly affecting neuronal plasticity [292], and melatonin is well known for its protective function against DNA damage [293] by increasing the DNA repair capacity [294].

One important question is whether melatonin can convey prophylactic effects against AD *via* its beneficial action on sleep. One of the elements of the answer could lie in the fact that circadian fluctuations of Aβ concentrations in the interstitial brain fluid and in the cerebrovascular fluid are thoroughly affected by sleep disorders by influencing the glymphatic brain system and Aβ production [295]. Considering that pineal melatonin is released directly into the CSF, its functional regulatory role in the neural glymphatic network and its physiological and therapeutic significance for neurocognitive health are well supported by experimental evidence indicating that melatonin is involved in clearing pathogenic toxins, like Aβ, from the brain to protect against neurocognitive decline [296]. Apart from other regions, sleep quality significantly also affects Aβ accumulation in the precuneus area of the brain, while sleep disturbance in AD is associated with a reduction of the volume of the precuneus that is functionally involved in memory [297]. Considering the importance of this brain area in AD, it is interesting to note that melatonin controls the activation of the precuneus [298].

Along the same lines, a cross-sectional study of healthy and cognitively unimpaired participants revealed a significant decrease in Aβ levels by increasing the night time sleep by just one hour [299], which suggests that a longer duration of sleep throughout life could reduce the risk of developing AD. Another PET imaging analysis combined with sleep polysomnography of clinically normal older adults stated that measuring sleep efficiency could predict the progression of Aβ deposition prior to the onset of cognitive symptomology in AD [300]. As a whole, considering that sleep has important roles in learning and memory consolidation and that its deprivation has a significant impact on the symptoms and progression of AD [301] and that melatonin augments total sleep time and decreases sleep onset latency, thus improving the overall quality of sleep [302], it seems reasonable to assume that this hormone may, *via* its sleep-enhancing activity, act as a prophylactic against AD.

Finally, beyond its effects on hippocampal neurogenesis, it is important to also consider the impact of melatonin on synaptogenesis. Indeed, this hormone has been shown to prevent the reduction in synaptogenic processes during the cerebellar development of chick embryos under excitotoxic neuronal degenerative conditions [303]. Furthermore, it has been demonstrated that melatonin exposure in high-fat diet fed rats prevents the reduction in synaptogenesis [304] and that maternal melatonin exposure has been shown to restore the delayed synaptogenesis in a rat model of intrauterine cortical dysplasia [305]. In addition, it has been revealed that melatonin supplementation improvised synaptogenesis in the olfactory bulbectomized rat [306] that has been proposed as an animal model of depression. Mechanistically, it has been evidenced that high intracellular cAMP levels tone down neuritogenesis and synaptogenesis during vertebrate neurodevelopment and that melatonin plays a significant role in downregulating cAMP levels [307]. As a whole, recent research has consistently supported the notion that melatonin contributes to neuronal survival, proliferation, and differentiation, such as dendritogenesis and axogenesis [308].

## 8 THE CONFRONTATION BETWEEN MELATONIN AND BENZODIAZEPINES WITH REGARD TO THE THERAPEUTIC TREATMENT AIMED AT CORRECTING THE DEFECTS OF THE CIRCADIAN RHYTHM

Although benzodiazepines (BZDs) are psychoactive drugs usually prescribed for circadian misalignment and insomnia, these medications produce side effects and significantly decrease memory function [309]. Furthermore, long-term use of BZDs is associated with an increased risk of cognitive impairment and dementia [310]. In addition, BZDs affect neurogenesis *via* GABA receptors and have been shown to cause neuronal apoptosis in the developing rodent brain [311]. Indeed, these drugs have complex effects on mitotic cells, which closely depend on the duration of exposure since long-term exposure reduces overall neurogenesis while short-term treatment increases proliferation [312]. Moreover, high concentrations of BZDs lead to the inhibition of DNA synthesis [313]. That melatonin can both enhance DNA repair capacity by regulating several key genes involved in DNA damage responsive pathways [294] and convey antiproliferative effects through a reduction of DNA synthesis [314] clearly illustrates the low versus high dosage response under different pathological conditions. This might be the case with BZDs also, but the side effects and toxicity of BZDs can probably lead to morbidity and mortality for some patients where the elderly are especially prone to toxicity [315], which is certainly not the case with melatonin administration, which is well acknowledged for least observed side and toxic effects. In this context, several studies have suggested that melatonin might be more suitable and effective than BZDs in the management of circadian misalignment and sleep disorders because melatonin improves sleep without significant performance impairment [316-318] and enhances neurogenesis and modulates the cell cycle machinery without toxic effects.

## CONCLUSION

**Fig. (2):**
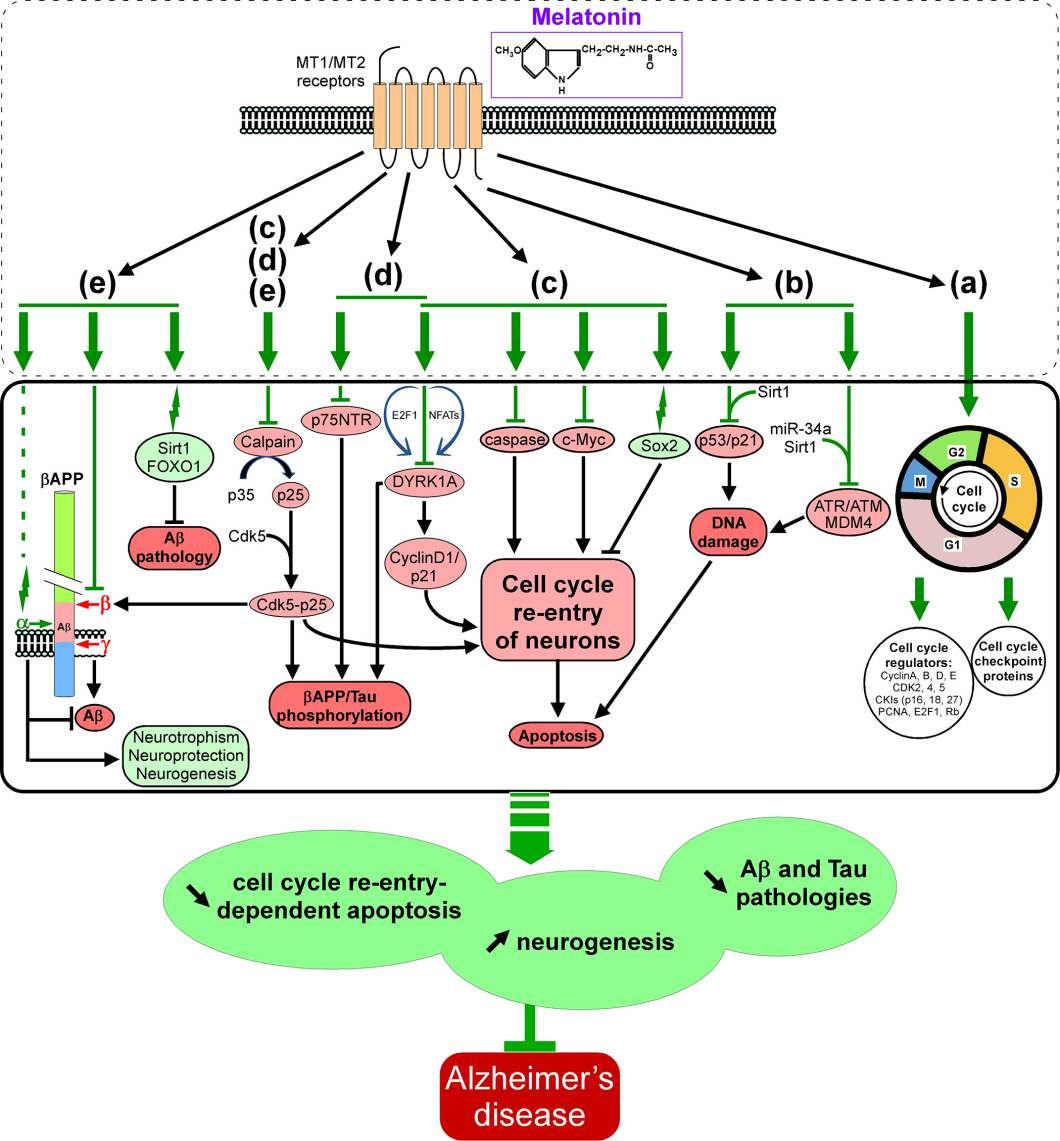
Schematic representation of the signalling pathways involved in melatonin-dependent regulation of the cell cycle, apoptosis and Aβ/tau pathologies. Alteration in the levels of cell cycle regulators results in aberrant cell cycle re-entry and subsequent apoptosis, contributing to AD progression. Melatonin, through its binding to the plasma membrane-located G-protein-coupled MT1 and MT2 receptors, coordinates and balances the cell cycle regulators (cyclins, CDKs, CKIs, E2F-1, Rb, and PCNA) involved in functions such as neuronal migration, neuronal maturation, and synaptic plasticity and regulates neuronal cell cycle checkpoint proteins (**a**). Moreover, melatonin controls the expression of DNA damage response genes and significantly prevents DNA damage-induced apoptosis *via* a sirtuin-dependent ATM/ATR/MDM4 and p53/p21 inhibition (**b**). Melatonin also has an important role in repressing several factors promoting cell cycle re-entry-dependent apoptosis (C-Myc expression, caspase activity, DYRK1-mediated cyclin D1/p21 levels and calpain-dependent conversion of p35 into p25) and in promoting Sox2 expression, which alleviates this phenomenon (**c**). In parallel, melatonin, by inhibiting DYRK1, p75NTR and by increasing Cdk5 activity *via* p25, is lessening βAPP and Tau hyperphosphorylation (**d**). In addition, melatonin, *via* its impact on βAPP processing (inhibition of β- and γ-secretases and activation of α-secretase as well as stimulation of the Sirt1/FOXO1 pathway), modulates Aβ production and promotes sAPPα-induced neuroprotection and neurogenesis (**e**). Finally and interestingly, melatonin, *via* its capability to inhibit calpain activity and the subsequent formation of the Cdk5/p25 hyperactive complex, has a triple beneficial effect by preventing cell cycle re-entry-dependent apoptosis (**c**), Cdk5/p25-dependent Tau hyperphosphorylation (**d**) and Cdk5/p25-mediated β-secretase activation and Aβ production (**e**). Overall, these multiple melatonin-induced beneficial effects altogether lead to a decrease in cell cycle re-entry-dependent apoptosis and Aβ and Tau pathologies as well as to an increase in neurogenesis and potentially represent a valuable way to thwart Alzheimer’s disease pathogenesis.

AD treatment and associated therapeutic interventions could be effective, but the lack of timely diagnosis impacts the overall prognosis and cost-effectiveness. It has been well documented that fluctuations in the circadian rhythms and cell cycle alterations are parts of early neurodegenerative processes, which likely correspond to diminished melatonin levels, thus further enhancing the ability of early diagnosis. Intriguingly, circadian and cell cycle-oriented interventions have been proposed as treatments for neurological disorders like AD. Melatonin not only regulates the circadian rhythm alterations but also prevents disrupted cell cycle-mediated variations and enhances adult neurogenesis (Fig. **2**). The insights provided in the present review would certainly aid in our understanding of the importance of optimizing the timing of AD-dementia diagnosis, which would potentially improve the prospects for melatonin intervention.

## Figures and Tables

**Table 1:** The factors targeted by melatonin to harmonize the cell cycle (reference are listed in order of appearance in the text).

**Dose**	**Duration**	**Model**	**Effect**	**References**
1 μM	12 h (pre incub)	Aβ_42_-stressed & H_2_O_2_-stressed human neuroblastoma cells	**↘ p75NTR** protein & mRNA levels	[142] [143]
1 mM 10 mg/kg/d	12 h (with MPP^+^) 9 m	MPP^+^-treated rat cerebellar granule cells 1 month-old SAMP8 mice	**↘ Cdk5** protein levels	[154] [156]
1 mM 10 mg/kg/d	12 h (with MPP^+^) 9 m	MPP^+^-treated rat cerebellar granule cells 1 month-old SAMP8 mice	**↘ p35 ➬ p25** conversion	[154] [156]
1 mg/kg/d & 0.1 mg/kg/d	7 d	Haloperidol-treated 5 month old rats	**↘ Cdk5** activity in hippocampus and cerebral cortex	[155]
1 nM 4 mM 1 mM	24 h (with E_2_) 4-48 h 24-48 h	Estrogen-treated human mammary adenocarcinoma cells Human osteosarcoma cells Human osteoblastic cells	**↘ Cyclin D1** transcription & protein levels	[157] [158] [161]
4 mM 1 mM	4-48 h 24-48 h	Human osteosarcoma cells Human osteoblastic cells	**↘ Cyclin B1** protein & mRNA levels	[158] [161]
4 mM 1 mM	24-48 h 4-48 h	Human osteosarcoma cells Human osteoblastic cells	**↘ Cdk4** protein & mRNA levels	[158] [161]
4 mM 1 mM	4-48 h 24-48 h	Human osteosarcoma cells Human osteoblastic cells	**↘ Cdk1** protein & mRNA levels	[158] [161]
250 μM	1 h pre incub + 24 h with DEX	Dexamethasone-treated human neuroblastoma cells	**↘ Calpain** activity & protein levels	[160]
250 μM	1 h pre incub + 24 h with DEX	Dexamethasone-treated human neuroblastoma cells	**↘ Calpastatin** protein levels	[160]
250 μM 20 mg/kg/d	15 d 14 d (with ATR)	Aged rat cerebellar granule neurons Splenocytes of atrazine-treated mice	**↘ E2F-1** protein levels	[136] [170]
0.25-4 mM	1 h (during activation)	Activated mouse CD4^+^ T cells	**↘ NFATs** activation (de-phosphorylation)	[173]
10-100 μg/ml	24 h	VBGP rat chondrocytes	**↘ PCNA** protein levels	[180]
1 mM	3 h (with Eto)	Etoposide-treated mouse oocytes	**↘ ATM** activation (phosphorylation)	[182]
10 mg/kg 3 d/week	6 w (just before BPA)	Cerebral neurons of bisphenol-A-treated rats	**↘ p53** expression levels	[187]
10 mg/kg/d 1 μM	6 m (from 16 to 22 m) 24h	Aged mice Aged human periodontal ligament stem cells	**↘ p53** protein & mRNA levels **↘ p53** protein levels	[191] [212]
1-10 mg/d	4 w	Aged SAMP8 mice	**↗ FoxO3A** mRNA levels	[190]
10 mg/kg/d	6 m (from 16 to 22 m)	Aged mice	**↗ FoxO1** mRNA levels	[191]
8 mg/kg/d	21 d (9 d before 5-FU + 12 d with 5-FU)	Hippocampus of 5-fluorouracil-treated rats	**↘ p21** positive cells	[211]
1 μM	24 h	Aged human periodontal ligament stem cells	**↘ p21** protein levels	[212]
3 mM	60 min (before H_2_O_2_)	H_2_O_2_-treated rat hepatoma cells	**↘ Ras** activation (Ras-GTP levels)	[214]
0.01-1 μM	1 h pre incub + 24 h with Aβ_42_	Aβ_42_-treated human neuroblastoma cells	**↘ APP-BP1** protein levels	[223]
0.1 μM	1 h pre incub + 24 h with Aβ_42_	Aβ_42_-treated human neuroblastoma cells	**↘** alteration of **NEDD8** cellular localization	[223]

**Abbreviations:** 5-FU, 5-fluorouracil; Aβ, amyloid beta; APP-BP1, amyloid precursor protein-binding protein 1; ATM, ataxia telangiectasia mutated; ATR, atrazine; BPA, bisphenol-A; Cdk, cyclin-dependent kinase; d, day; DEX, dexamethasone; E_2_, estradiol; Eto, etoposide; Fox, forkhead box; GTP, guanosine-5'-triphosphate; h, hour; m, month; min, minute; MPP^+^, 1-methyl-4-phenylpyridinium; NEDD, neural precursor cell expressed developmentally downregulated; NFAT, Nuclear factor of activated T-cells; p75NTR, p75 neurotrophin receptor; PCNA, proliferating cell nuclear antigen; Ras, rat sarcoma virus; SAMP, senescence accelerated mouse-prone; VBGP, vertebral body growth plate; w, week.

**Table 2:** The factors targeted by melatonin to promote adult neurogenesis (reference are listed in order of appearance in the text).

**Dose**	**Duration**	**Model**	**Effect**	**References**
8 mg/kg/d 3 mg/kg/d (d1-d3) 5 mg/kg/d (d4-d3) 10 mg/kg/d (d6-d7) 6 mg/L (amount/d NI)	21 d (9 d before 5-FU + 12 d with 5-FU) 7 d 3 w (7 w after D-gal)	Hippocampus of 5-fluorouracil-treated rats Adult mouse hippocampus Dentate gyrus of D-gal-treated mice	**↗ DCX** protein levels	[211] [258] [271]
8 mg/kg/d 10 mg/kg/d 10 mg/kg/d 5 mg/kg/d	21 d (9 d before 5-FU + 12 d with 5-FU) 6 m (16 to 22 m old) 8 w 9 w + exercise training	Hippocampus of 5-fluorouracil-treated rats Hippocampus and prefrontal cortex of aged mice Hippocampus of non-obese and obese mice Cerebellum of high-fat diet-fed rats	**↗ BDNF** protein levels	[211] [275] [276] [277]
1-3 mM 100 nM	24 h 2-3 d	C6 glioma cells Mouse neural stem cells	**↗ Sox2** mRNA levels	[250] [251]
1-4 μM	24 h	Human neuroblastoma cells	**↗ β-catenin** protein levels	[256]
3-5 mM 3 mg/kg/d (d1-d3) 5 mg/kg/d (d4-d3) 10 mg/kg/d (d6-d7)	24 h 7 d	C6 glioma cells Adult mouse hippocampus	**↗ Nestin** mRNA levels **↗ Nestin** protein levels	[250] [258]
3 mg/kg/d (d1-d3) 5 mg/kg/d (d4-d3) 10 mg/kg/d (d6-d7)	7 d	Adult mouse hippocampus	**↗ β3-tubulin** protein levels	[258]
10 nM-100μM	4 d	H_2_O_2_-treated human mesenchymal stem cells	**↗ SIRT1** mRNA levels	[261]
6 mg/L (amount/d NI)	3 w (7 w after D-gal)	Dentate gyrus of D-gal-treated mice	**↗ Ki67**protein levels	[271]

**Abbreviations:** 5-FU, 5-fluorouracil; BDNF, brain-derived neurotrophic factor; d, day; DCX, doublecortin; D-gal, D-galactose; h, hour; L, liter; m, month; NI, non indicated; SIRT1, sirtuin 1; Sox2, SRY (sex determining region Y)-box 2; w, week.

## LIST OF ABBREVIATIONS

AD: Alzheimer’s Disease
Cdks: Cyclin-dependent Kinases
CKIs: Cyclin-dependent Kinase Inhibitors
CNS: Central Nervous System
HD: Huntington's Disease
MCI: Mild Cognitive Impairment
NSCs: Neural Stem Cells
PD: Parkinson’s Disease
RNS: Reactive Nitrogen Species
ROS: Reactive Oxygen Species
SCN: Suprachiasmatic Nucleus
SGZ: Sub Granular Zone
UPS: Ubiquitin-proteasome System
